# Varying effects of chlorination on microbial functional repertoire and gene expression in contrasting effluents

**DOI:** 10.3389/fmicb.2025.1593147

**Published:** 2025-06-18

**Authors:** Mandy Lok Yi Tang, Stanley Chun Kwan Lau

**Affiliations:** Department of Ocean Science, Hong Kong University of Science and Technology, Kowloon, Hong Kong SAR, China

**Keywords:** metagenomics, metatranscriptomics, sewage effluents, chlorination, microbiomes, functional genes, gene expression

## Abstract

Effluents produced from different influent sources and sewage treatment processes carry distinct microbial community compositions. These microbiomes exhibit varying degrees of resistance and resilience under chlorination; however, their survival strategies and potential risks to the public health and ecosystem have yet to be fully characterized. In view of this, we subjected microbiomes from two contrasting types of effluents with distinct influent properties (seawater/freshwater-based) and prior treatment processes (primary/secondary) to metagenomics and metatranscriptomics analyses for comparing the alterations in their functional genes and activities under chlorination. The effluents presented highly dissimilar genomic and transcriptomic profiles. The variations in these profiles were significantly correlated to physicochemical factors including salinity, DO, BOD₅, TSS, and TN. We recovered novel metagenome-assembled genomes (MAGs) from each type of effluent, revealing that those recovered from the same effluent tended to share similar functional properties which aligned with the physicochemical parameters of the effluent. Notably, the type and extent of alterations in genomic and transcriptomic profiles under chlorination varied greatly between effluents. Most of the genes and transcripts with significant changes in relative abundances were exclusive to their respective effluents. Also, the number of genes and transcripts with significant increase in relative abundances after chlorination were much higher than those with reduction. These enriched genes and transcripts were responsible for a wide range of functions, including energy generation, repair of damaged components and stress responses. Furthermore, the remanent microbiomes in chlorinated effluents still harbored numerous genes related to waterborne diseases and antimicrobial resistance, suggesting the potential risks of discharging these effluents into the environment. This study revealed the diverse effects of chlorination on different types of effluent microbiomes. It suggested that the remanent microbiomes in chlorinated effluents would have great variance in genetic potential and activities, providing insights into the evaluation and regulation of chlorine disinfection in sewage treatment.

## 1 Introduction

Chlorination has been widely used for sewage disinfection since the late 19th century due to its strong microbiocidal effect, low cost and rapid reaction. During chlorination, reactive chlorine species (RCS) attack and permeate microbial cell membranes, oxidizing a wide range of vital cellular components (Virto et al., 2005; Fukuzaki, 2006; Mizozoe et al., 2019). The extensive damage of biomolecules disrupts essential physiological functions and cellular homeostasis, ultimately resulting in cell death.

However, the survival of microbes under chlorine stress can be influenced by many external environmental factors. Previous research showed that coliform bacteria isolated from effluents, river and biofilms in distribution systems exhibited different degrees of sensitivity to chlorine (Sartory and Holmes, 1997). Some abiotic factors such as the level of organic matter determine the chlorine demand of medium and thus the amount of RCS acting on the microbial cells. Organic matters can also stabilize cellular structures against permeabilization, especially the outer membranes of gram-negative bacteria (Virto et al., 2005). Other factors such as attachment to inert surfaces, prior growth in low-temperature or nutrient-rich conditions, and high pH can promote the formation of extracellular polymeric substances (EPS), which act as protective barriers against chlorine attack (Stewart and Olson, 1992; Ryu and Beuchat, 2005; Yang et al., 2016).

Apart from environmental conditions, microbial sensitivity to chlorine also varies among species due to their inherent structural and physiological characteristics. Gram-positive bacteria generally exhibit higher chlorine resistance than gram-negative bacteria because of their thicker peptidoglycan cell walls (Mir et al., 1997; Virto et al., 2005). Spores, with their thick protein coat, are highly resistant to chlorine as well (Zeng et al., 2020). Studies also showed that some chlorine-resistant strains have higher cellular fatty acid composition and EPS compared to the sensitive strains (Furuhata et al., 2014; Wang et al., 2019). These structural properties provide microbes with physical and chemical barriers against chlorine attack. Moreover, some microbes have the ability to activate various cellular responses to cope with chlorine-induced oxidative stress, such as activating chaperones to prevent protein aggregation and upregulating the expression of reductase to repair oxidized molecules (Gray et al., 2013; Nizer et al., 2020). These stress responses can alleviate the damage caused by chlorine and increase their chance of survival.

In sewage treatment works, domestic sewage is a complex mixture consisted of varying amounts of organic and inorganic matters. The effluents in different treatment works can have very distinct physicochemical parameters and microbial community compositions due to different sources of influents and types of treatment processes. In our previous works, we observed that the impact of chlorination on microbes varied greatly between different effluents, including the enriched or diminished bacterial populations and their survival after releasing from chlorine stress (Tang and Lau, 2022, 2023). The microbiomes appeared to have large variations in the strength of resistance and resilience to chlorine stress, which would affect the disinfection efficiency of chlorine and microbiological safety of effluent discharge. Yet, investigations on the features of microbial survival mechanisms and activities under chlorination in different types of effluents are limited. The alterations in genomic and transcriptomic profiles of microbiomes after chlorination are important for unraveling their strategies to overcome the chlorine stress. Such information can also help predict the adaptation and potential threats of the remanent microbiomes in chlorinated effluents, providing valuable insights for the evaluation and regulation of chlorine disinfection and sewage discharge.

In this study, metagenomics and metatranscriptomics analyses were utilized to explore the changes of functional repertoire and gene expression profiles of the microbiomes before and after chlorination in two contrasting types of treated effluents. One of the effluents is seawater-based sewage with primary treatment. The other one is freshwater-based sewage with secondary treatment. Both effluents undergo chlorination before discharge to the receiving waters. Based on the amplicon sequencing results from previous study, there was a large discrepancy between the bacterial taxonomic compositions of the two effluents (Tang and Lau, 2022). The alterations in compositions under chlorination varied between effluents. To expand upon this understanding, the aim of this study is to investigate the survival strategies and stress responses of different effluent microbiomes under the impact of chlorination. Metagenome-assembled genomes (MAGs) were also recovered from each effluent to examine the evolutionary relationships and genetic functions of microbes. The potential risks associated with the genomic potential and activities of the remanent microbiomes in chlorinated effluents will be discussed.

## 2 Materials and methods

### 2.1 Experimental design

The effluent samples investigated in this study were selected from our previous one-year bimonthly sampling of pre- and post-chlorination effluents from two sewage treatment works with contrasting properties (Tang and Lau, 2022). In that study, effluents were collected from two sewage treatment works before and after chlorination. During each sampling event, triplicates effluent samples were collected in 5-min intervals at the upstream of chlorine injection (pre-disinfection) and downstream dechlorination (post-disinfection). The time lapse between the collection of pre- and post-disinfection samples was 20 min, which is the approximate time required for the effluents to travel through the chlorine mixing chamber. The samples were transferred into autoclaved polypropylene (PP) bottles and added with an excess of 0.1 M Na_2_S_2_O_3_ to exhaust any remaining chlorine. Temperature, salinity, dissolved oxygen (DO), pH, turbidity of the samples were measured on site using a multiparameter water quality sonde (YSI, United States). The biological oxygen demand (BOD_5_), total suspended solids (TSS), total nitrogen (TN), ammonia (NH_3_), nitrite (NO_2_^−^), nitrate (NO_3_^−^), total phosphorous (TP), phosphate (PO_4_^3−^), and silicate (SiO_3_^2−^) were measured in the laboratory according to APHA methods (APHA, 2005).

Based on the results of amplicon sequencing, samples collected in 3 months (August 2019, December 2019, and April 2020) exhibited large variations in the taxonomic compositions, with significant difference tested by PERMANOVA (*p* < 0.001, *R*^2^ = 0.57). These samples were chosen for metagenomics and metatranscriptomics analyses in this study to capture the variance of effluents, so as to reveal a range of effects that chlorination imposed to the microbiomes.

The DNA or RNA obtained from the triplicates of pre-/post-chlorination effluents of each month was combined for sequencing because the variability in their taxonomic compositions were relatively low based on the amplicon sequencing results (Tang and Lau, 2022). The differences between replicates were not significant (e.g., pre-chlorination replicates collected in December: *p* = 0.24, PERMANOVA). Meanwhile, the biological replicates (i.e., pre-/post-chlorination effluents in 3 months) were retained for the comparative analyses of pre- and post-chlorination microbiomes in each sewage treatment works.

### 2.2 Sewage treatment works

The two sewage treatment works involved were Stonecutters Island Sewage Treatment Works (SC) and Stanley Sewage Treatment Works (ST) in Hong Kong (Supplementary Figure S1). SC is one of the largest primary sewage treatment plants in the world with a design daily flow of 2.45 million m^3^, serving a population of 5.7 million. It receives around 1.9 million m^3^/d of seawater-based municipal sewage from coastal regions (areas around the Victoria Harbour, Kowloon and part of Hong Kong Island) which use seawater for flushing. Chemically enhanced primary treatment (CEPT) is applied in SC for coagulation, flocculation and sedimentation processes. During the CEPT, ferric chloride and polymer are mixed with the sewage inflow. After flocculation and sedimentation, the sludge and scum are dewatered and removed. The treated sewage will then undergo chlorination before discharge. In contrast, the treatment capacity of ST is relatively small scale, with a design daily flow of 11.6 k m^3^. It collects around 9 k m^3^/d freshwater-based municipal sewage from southern part of Hong Kong Island. The distance between SC and ST is about 14.9 km. In ST, coarse suspended solids are first removed by screening and degritting. The sewage then goes through secondary treatment, in which oxygen is fed to the aeration tanks for the growth of microbes to assimilate the pollutants. The retention time is about 15 h. After that, the activated sludge and scum are removed from the effluent at sedimentation tanks. The treated effluents will then undergo chlorination. Both sewage treatment works use sodium hypochlorite solution for disinfection before discharging the effluents to coastal seawater. The average dosage of sodium hypochlorite used in SC was 16.3 mg/L, while that in ST was 3 mg/L. The contact time is around 20 min in both plants. The primarily treated effluents in SC or secondarily treated effluents in ST before and after chlorination were used in this study.

### 2.3 DNA and RNA extraction

SC and ST effluent had large differences in total bacterial loads, the concentration of total bacteria in SC was around 100 times higher than that in ST, as shown by the quantitative PCR (qPCR) in previous study (Tang and Lau, 2022). Moreover, the bacterial communities in ST effluents had higher richness and lower evenness comparing to SC effluents, reflected by the alpha-diversity indices in previous study. Therefore, to achieve comparable amounts of DNA and RNA, and compensate for the disparity of diversity between effluents, the sample volumes required for ST samples were higher than that of SC samples. The volumes of SC samples used for extraction were 100 mL each, while that of ST samples were 6 and 8 L before and after chlorination, respectively. The sample volumes were determined according to the yields of extracted DNA and RNA in preliminary tests.

Each effluent sample was first dispensed into 250 mL tube and centrifuged at 12,000 rpm for 10 min to obtain cell pellets. The process was repeated until the required sample volume was reached. The cell pellet was then washed with autoclaved phosphate-buffered saline (PBS), centrifuged and resuspended in 1 mL RNA*later* (Sigma-Aldrich), then stored at −80°C to preserve the cellular RNA. Before DNA and RNA extraction, the RNA*later* was removed by centrifugation at 15,000 rpm for 5 min and discarding the supernatant. The DNA and RNA were extracted simultaneously using AllPrep^®^ DNA/RNA Mini Kit (Qiagen) following the manufacturer’s instructions. As the triplicates of pre-/post-chlorination effluents of each month was combined after extraction, there were 12 DNA and 12 RNA samples in total (2 sewage treatment works, 3 months of sampling, before chlorination and after chlorination).

### 2.4 Sequencing and data processing

For the library preparation, the metagenomic DNA was first randomly fragmented by sonication and then end-repaired, A-tailed and ligated with adapters. After PCR amplification and purification, the DNA libraries were checked for size distribution by Agilent 2100 Bioanalyzer (Agilent Technologies, CA, United States). For the total RNA, rRNA was first removed using Ribo-Zero kit. The remaining mRNA was then randomly fragmented and used as the templates for cDNA reverse transcription. Same as the processing of DNA, the cDNA went through end repair, ligation, PCR amplification and quality checking processes to construct the libraries for sequencing.

The DNA and RNA libraries were sequenced on Illumina NovaSeq 6000 platform to generate 20 million 150 bp paired-end reads each for the metagenomics and metatranscriptomics. Raw reads with adapter contamination, > 10% of uncertain nucleotides (“N” base calls) or > 50% of low quality nucleotides (base quality score less than 5) were discarded. The quality of reads was maintained above Q20. Read assembly, 16S rRNA taxonomic classification (any remaining rRNA reads in transcriptomes were also identified), ORF prediction, functional assignments (KEGG Orthology), read mapping and functional pathway prediction were performed using the coassembly mode of SqueezeMeta v1.6.0 pipeline (Tamames and Puente-Sánchez, 2019). Pre-/post-chlorination samples from the same sewage treatment work were co-assembled to generate larger reference collection of contigs. Reads from individual samples were then mapped back to the co-assembly.

For the binning processes, short contigs with < 200 bps were removed. The completeness and contamination of bins were verified by the CheckM software v1.1.6 (Parks et al., 2015). Only MAGs with > 90% completeness and < 10% contamination were selected for further analyses. The taxonomies of these MAGs was classified against Genome Taxonomy Database (GTDB) r214 using GTDB-tk v2.3.2, with a species boundary cut-off of 95% Average Nucleotide Identity (ANI) value (Chaumeil et al., 2020; Parks et al., 2022).

### 2.5 Statistical analyses

Differential expression (DE) analysis was used to identify significant differences between two effluents as well as pre- and post-chlorination by using edgeR in R software v 4.2.1 (Robinson et al., 2009). Trimmed Mean of the M-values (TMM) normalization was applied for all mapped read counts of genes and transcripts to account for the bias from the sequencing depth and some highly expressed genes. Quasi-likelihood F-test (QLF) was used to determine significantly different gene abundance or expression between sample groups (*p*-value < 0.05). EdgeR was applied for the metagenomic data as TMM normalization had robust performance on gene abundance counts for eliminating the between-sample variability of absolute DNA counts (Pereira et al., 2018). The consistency of analysis pipeline used for both differential abundance and expression analyses would also minimize the technical variations that might affect the comparability of the identified genes and transcripts.

The similarity of the genomic or transcriptomic profiles of samples shown in the multi-dimensional scaling (MDS) plots was measured based on the leading log_2_ fold change of TMM-normalized counts of genes or transcripts using edgeR. The leading log_2_ fold change was defined as the root-mean-square of the largest 500 log_2_ fold changes of genes or transcripts between each pair of samples. PERMANOVA test was carried out in R using adonis2 package to determine the correlations between physicochemical parameters and the genomic or transcriptomic profiles, significant correlations were determined by FDR of 5% (*q* < 0.05). The effects of station, chlorination and month on the genomic or transcriptomic profiles were also tested by PERMANOVA. Venn diagrams were constructed using the online software.^1^ The dot plots showing the functional pathways of genes and transcripts with significant increase in relative abundances after chlorination were constructed using Prism 9 software.^2^

For binning, the placement of MAGs in *de novo* phylogenetic tree was inferred from concatenated alignment of 120 marker genes using the de_novo_wf command of GTDB-tk and visualized with iTOL v6.8.2^3^ (Letunic and Bork, 2021). A MAG is regarded as novel if it does not have an ANI value of ≥ 95% with any MAG in the GTDB. The MAGs were then compared with Unified Human Gastrointestinal Genome (UHGG) v2.0.2 catalog to identify those that were likely to originate from the human gut microbiome using Mash v2.3 and calculate the percentages of matched k-mers using MGnify (Ondov et al., 2016; Almeida et al., 2021; Gurbich et al., 2023). The ANI values between MAGs were computed using OrthoANIu (Yoon et al., 2017).

## 3 Results

### 3.1 Genomic and transcriptomic profiles of the two types of effluents

Metagenomic and metatranscriptomics sequencing of the 12 samples yielded an average of over 6 × 10^7^ filtered reads per sample. All the reads had > 90% of the bases with quality score over 30 (Q30). The numbers of reads, Q30 values and GC contents are summarized in Supplementary Table S1.

**Figure 1:**
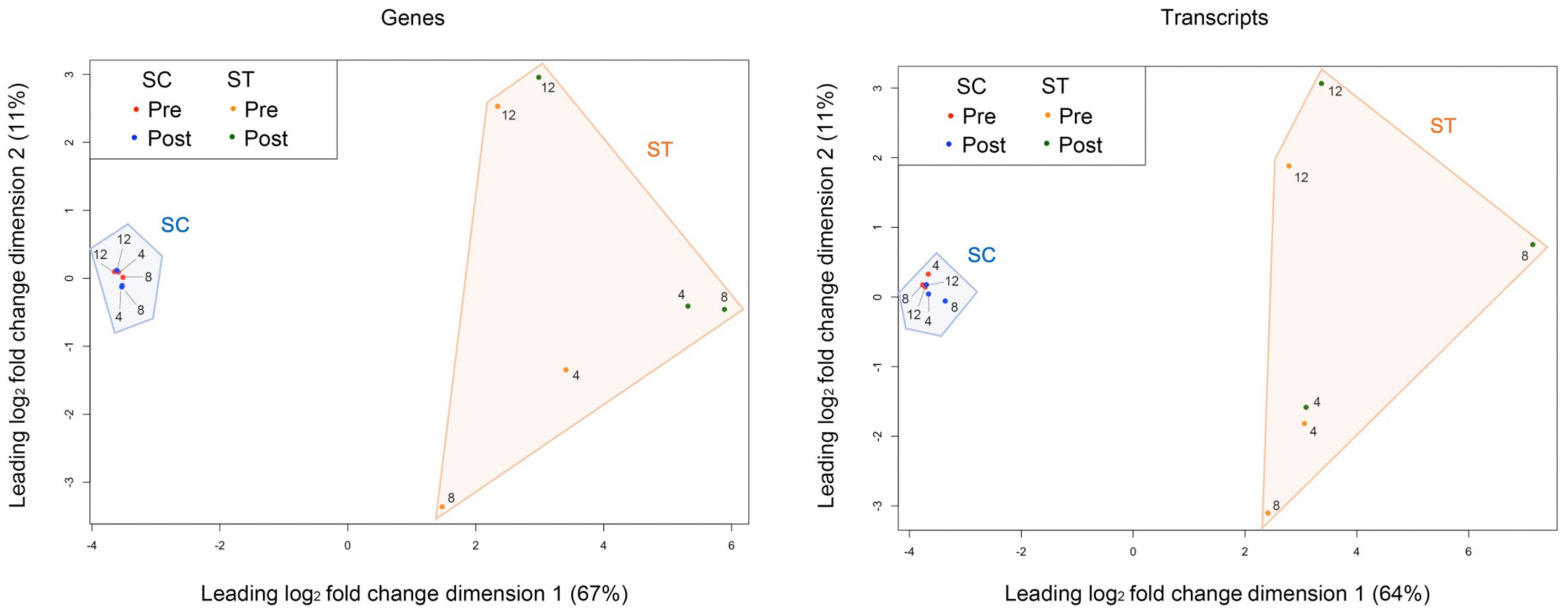
Multi-dimensional scaling (MDS) plots of the genomic and transcriptomic profiles. The similarity between the genomic or transcriptomic profiles of samples was measured based on the leading log_2_ fold change (root-mean-square of the largest 500 log_2_ fold changes) of TMM-normalized counts of genes or transcripts. The numbers 4, 8, and 12 represent the month of sample collection (i.e., Apr, Aug, and Dec). The percentage of total variance explained by each dimension is indicated on the axis. Pre and Post represent samples before and after chlorination, respectively.

The similarities of genomic or transcriptomic profiles between samples are shown in the MDS plots (Figure 1). Clear separation between the SC and ST effluent was observed along dimension 1 for both genomic and transcriptomic profiles, which accounted for over 60% of the total variance. The profiles of SC samples are closely clustered, showing high similarity between sampling months. In contrast, large variations existed in the profiles of ST samples between months. The variations between pre- and post-chlorination samples were also greater than that in SC samples.

**Figure 2:**
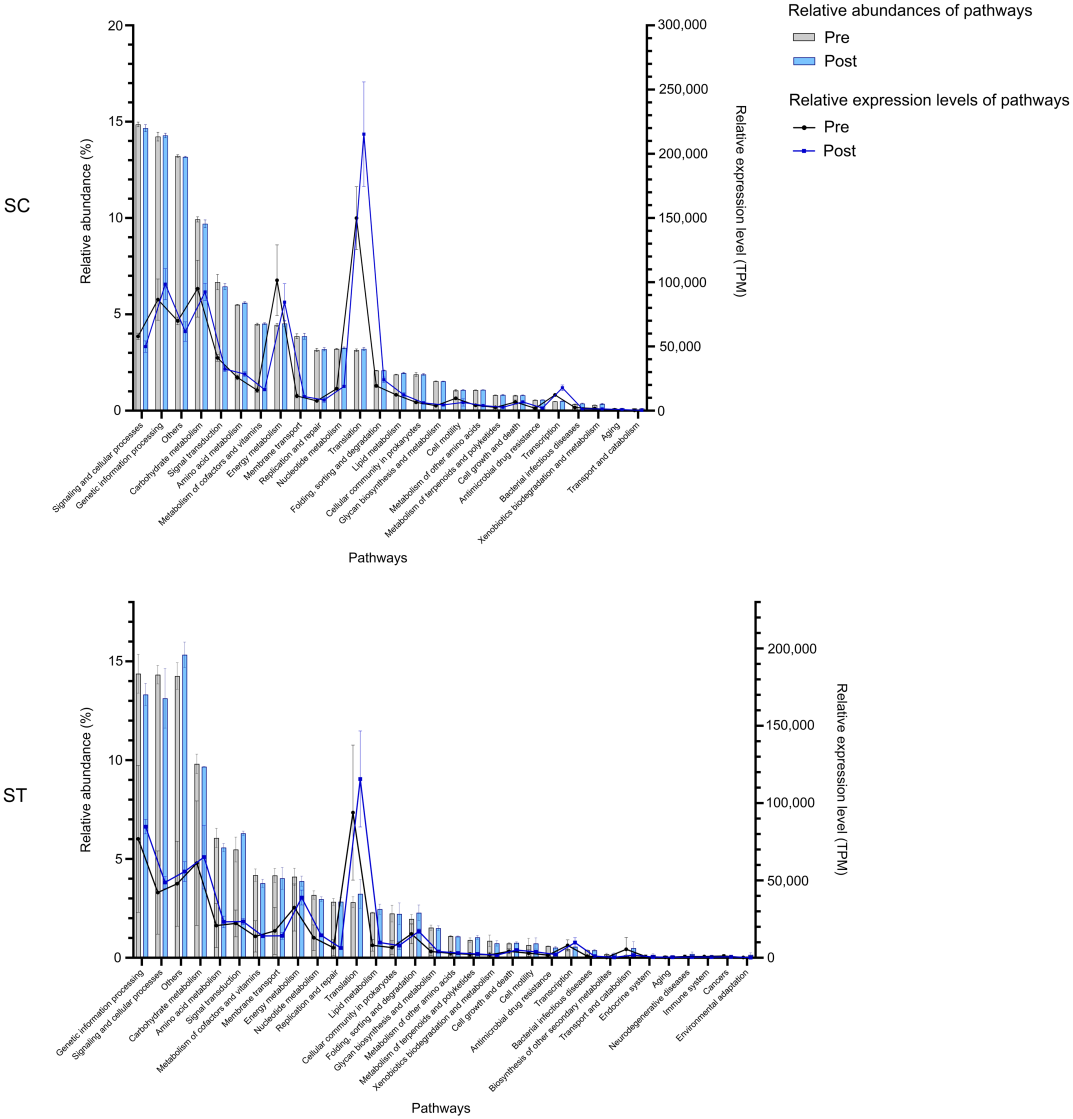
The relative abundances and expression levels of functional pathways in SC and ST effluents. The relative abundances of pathways were calculated by the percentages of DNA reads assigned to each pathway over the total mapped reads, while the relative expression levels of pathways were calculated by the numbers of RNA reads assigned to each pathway normalized by both gene length and total mapped reads. Pathways with relative abundance < 0.1% in all samples were excluded. Bars and lines indicate the relative abundance and expression of pathways, respectively. Error bars are shown as ± 1 S. D. Pre and Post represent samples before and after chlorination, respectively.

Comparing the percentages of DNA reads mapped to different functional pathways in two effluents, the two most abundant pathways in both effluents were signaling and cellular processes, and genetic information processing (Figure 2). Both pathways accounted to over 12% of the total mapped reads. Other abundant functional pathways including carbohydrate metabolism, signal transduction and amino acid metabolism were all over 5%. For RNA, the functional pathways with higher relative abundances or expression levels were also similar in SC and ST samples. Translation had higher expression level after chlorination in both effluents. Other highly expressed pathways in both effluents included energy metabolism, genetic information processing and carbohydrate metabolism. Besides, it is noted that the pathways in ST samples had greater variations before and after chlorination comparing to those in SC, particularly for the samples collected in August (Supplementary Figure S2).

The variations in genomic and transcriptomic profiles showed significant correlations with some of the measured physicochemical parameters (*q* < 0.05, PERMANOVA) (Table 1). Both genomic and transcriptomic profiles were significantly correlated to DO, salinity, turbidity, BOD_5_, TSS, and TN (*q* < 0.05). NH_3_ also showed significant correlation with the genomic profile but the proportion of variation being explained by this factor was relatively low based on the R^2^ value. In contrast, temperature, pH and other nutrient parameters including NO_2_^−^, NO_3_^−^, TP, PO_4_^3−^, and SiO_3_^2−^ did not have significant correlations with the genomic or transcriptomic profiles (*q* > 0.05).

**Table 1:** PERMANOVA test on the correlations between the variations of genomic or transcriptomic profiles and physicochemical parameters.

Profile	Statistic	Temperature	DO	Salinity	pH	Turbidity	BOD_5_	TSS	TN	NH_3_	NO_2_^−^	NO_3_^−^	TP	PO_4_^3−^	SiO_3_^2−^
Genome	*R* ^2^	0.053	**0.366**	**0.456**	0.127	**0.390**	**0.423**	**0.434**	**0.340**	**0.281**	0.260	0.108	0.094	0.166	0.113
	pseudo-*F*	0.559	**5.768**	**8.373**	1.455	**6.391**	**7.340**	**7.672**	**5.150**	**3.917**	3.516	1.211	1.042	1.985	1.268
	*q*-value	0.695	**0.007**	**0.007**	0.305	**0.008**	**0.007**	**0.007**	**0.021**	**0.028**	0.056	0.341	0.420	0.218	0.333
Transcriptome	*R* ^2^	0.065	**0.263**	**0.366**	0.078	**0.309**	**0.333**	**0.354**	**0.265**	0.211	0.115	0.112	0.080	0.164	0.129
	pseudo-*F*	0.696	**3.568**	**5.773**	0.845	**4.465**	**4.999**	**5.471**	**3.599**	2.681	1.301	1.265	0.873	1.963	1.476
	*q*-value	0.620	**0.031**	**0.014**	0.512	**0.028**	**0.019**	**0.014**	**0.033**	0.064	0.325	0.367	0.502	0.165	0.289

*Q*-values were obtained using 999 permutations of residuals under reduced model and FDR correction. *R*^2^ and pseudo-*F* values are listed in the table. Parameters with significant correlations (*q* < 0.05) are indicated in bold.

In addition, the effects of station, chlorination and month on the genomic and transcriptomic profiles were also tested by PERMANOVA. Both genomic and transcriptomic profiles were significantly correlated to the difference of sewage treatment plants (DNA: *R*^2^ = 0.464, *p* < 0.001; RNA: *R*^2^ = 0.367, *p* < 0.01). Regarding to the effect of chlorination in each station, the correlations with both genomic and transcriptomic profiles were significant (DNA: *R*^2^ = 0.653, *p* < 0.01; RNA: *R*^2^ = 0.515, *p* < 0.01). Significant correlations were also exhibited for different months in each station (DNA: *R*^2^ = 0.678, *p* < 0.05; RNA: *R*^2^ = 0.515, *p* < 0.01).

### 3.2 Alterations of genes and transcripts after chlorination

**Figure 3:**
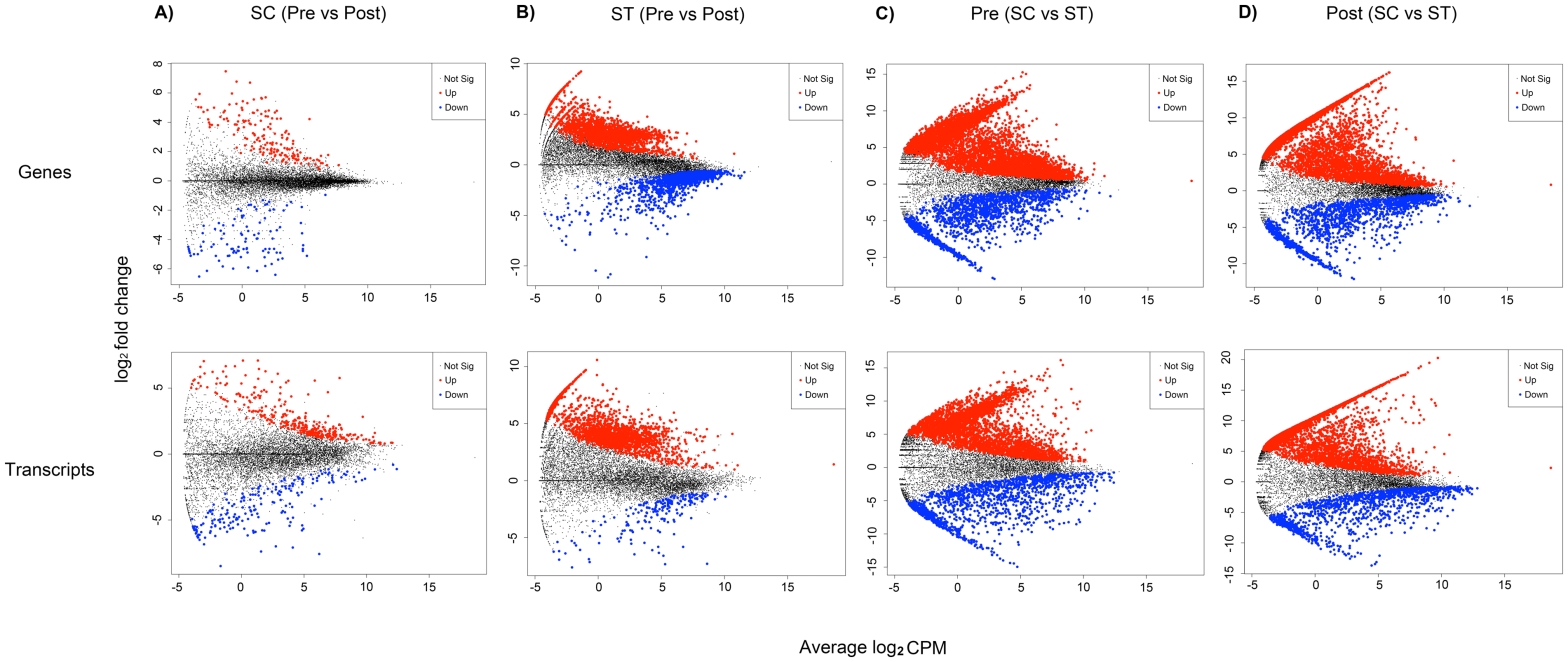
Genes and transcripts with significantly different relative abundances in DE analysis. These plots show the fold change against relative abundance of all mapped genes and transcripts in different sample groups of comparison. Quasi-likelihood F-test (QLF) were applied to determine genes and transcripts with significant different relative abundances. Four groups of comparison were shown: **(A)** samples before and after chlorination in SC; **(B)** samples before and after chlorination in ST; **(C)** pre-chlorination (Pre) samples in SC and ST; **(D)** post-chlorination (Post) samples in SC and ST. For **(A)** and **(B)**, genes and transcripts that had significantly higher relative abundances in the post-chlorination samples are highlighted in red, those with significantly lower relative abundances are highlighted in blue (*p*-value < 0.05). For **(C,D)**, genes and transcripts that had significantly higher relative abundance in ST samples are highlighted in red, those with significantly lower relative abundance are highlighted in blue (*p*-value < 0.05). Data points in black color are genes or transcripts with no significant difference. Log_2_ CPM is the log_2_ average of the counts per million reads.

DE analysis identified 279 genes and 527 transcripts with significant changes in relative abundances after chlorination in SC samples (*p* < 0.05, Quasi-likelihood F-test), accounting for 2.06 and 3.89% of the total mapped genes and transcripts (Figure 3A). For ST samples, the proportions of significantly changed genes and transcripts were much higher (Figure 3B). There were 3,742 genes and 2,162 transcripts with significant changes in relative abundances after chlorination, accounting for 27.59 and 15.94% of the total mapped reads. The genes and transcripts in ST samples also had greater fold changes compared to those in SC, especially for those with higher relative abundances after chlorination. For DNA, the log_2_ fold changes in ST reached 9 and −11 for upregulated and downregulated genes respectively, while that in SC were only 7 and −6, respectively. For RNA, the log_2_ fold changes in ST reached 10 and −7 for upregulated and downregulated genes respectively, while that in SC were 7 and −8, respectively.

Considering the pre- and post-chlorination samples separately, large proportions of the genes and transcripts were significantly different between the two types of effluents (*p* < 0.05, Quasi-likelihood F-test) (Figures 3C,D). Before chlorination, 53.18% of genes and 37.02% of transcripts had significantly different relative abundances between SC and ST samples. After chlorination, the proportions increased to 55.76 and 40.39%, respectively.

**Figure 4:**
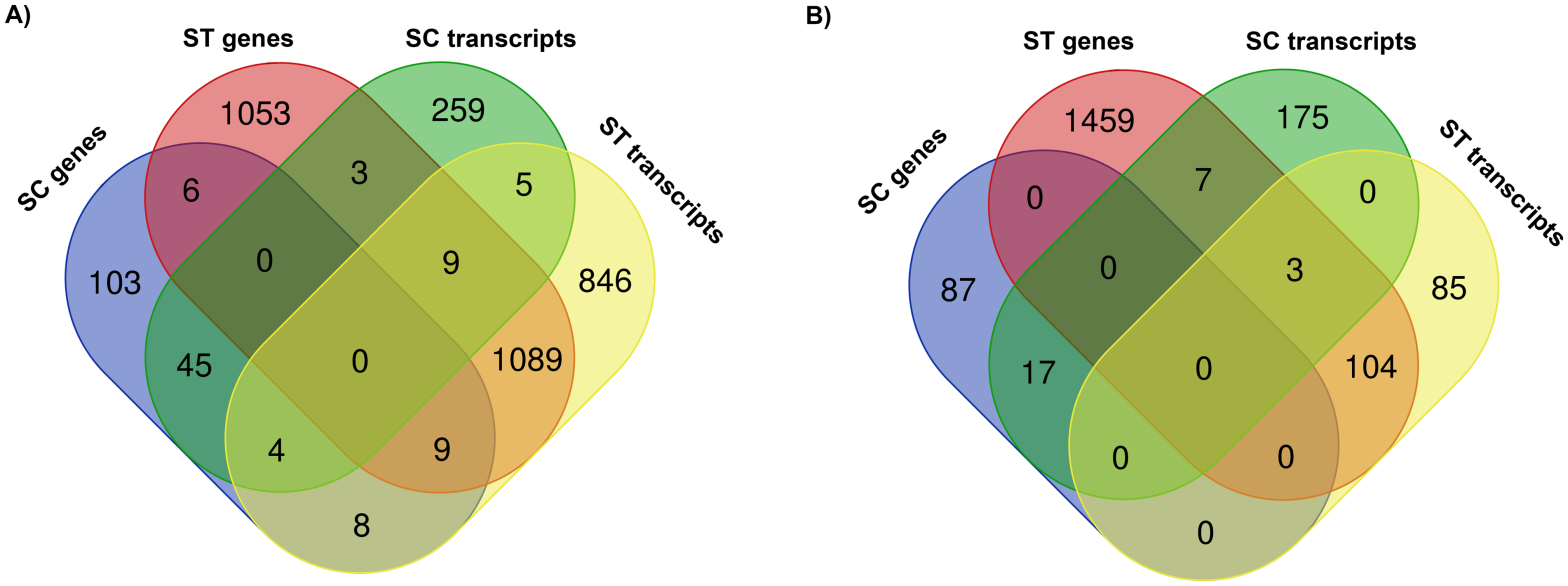
Venn diagrams showing the numbers of genes and transcripts with significant **(A)** increase or **(B)** decrease in relative abundances after chlorination in SC and ST (*p* < 0.05, Quasi-likelihood F-test). The overlapping circles indicate the numbers of genes or transcripts commonly found in the sample groups.

The numbers of genes and transcripts with significant change in relative abundance after chlorination are shown in the Venn diagrams (*p* < 0.05, Quasi-likelihood F-test) (Figure 4). Only a few number of genes and transcripts were found to be commonly increased in both types of effluents, they included different kinds of enzymes such as ubiquitin-protein ligase, serine/threonine-protein kinase and dimethylsulfide dehydrogenase (Figure 4A). None of the genes were commonly reduced in both effluents (Figure 4B). Only 3 transcripts were commonly reduced. Therefore, most of the significantly changed genes and transcripts were unique to the effluent type. Regarding to the genes which had significant changes in relative abundances of both DNA and RNA reads, there were 49 genes in SC and 1,107 genes in ST that had significant increase of both reads (Figure 4A), while 17 genes in SC and 107 genes in ST had significant reduction of both reads (Figure 4B).

### 3.3 Alterations of functional pathways after chlorination

**Figure 5:**
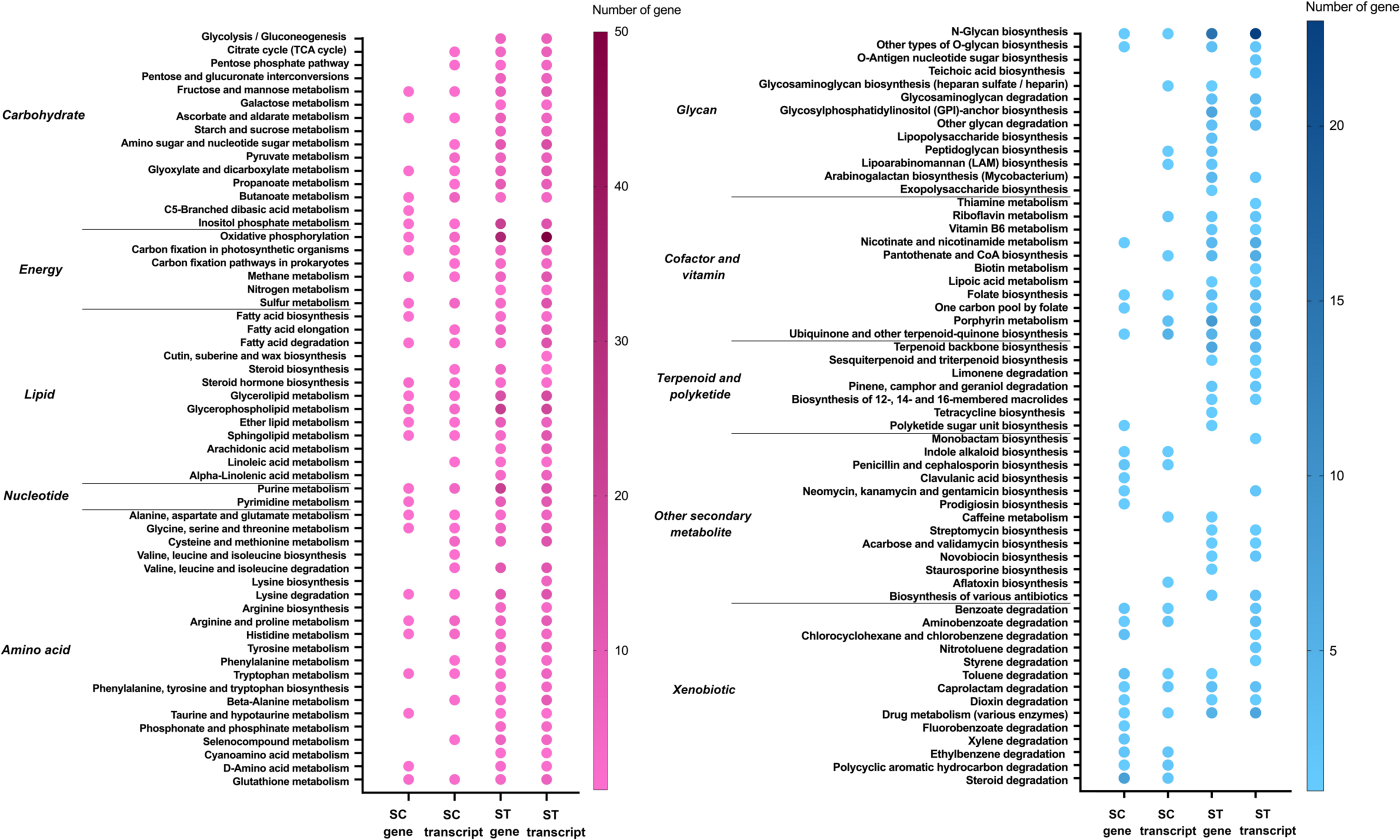
Metabolic pathways of genes and transcripts with significant increase in relative abundance after chlorination in SC or ST samples (*p* < 0.05, Quasi-likelihood F-test). Color scale indicates the number of gene or transcript mapped in that pathway. Absence of dot means that no gene or transcript of that pathway was enriched. The metabolic pathways are divided into two panels for clear visualization.

**Figure 6:**
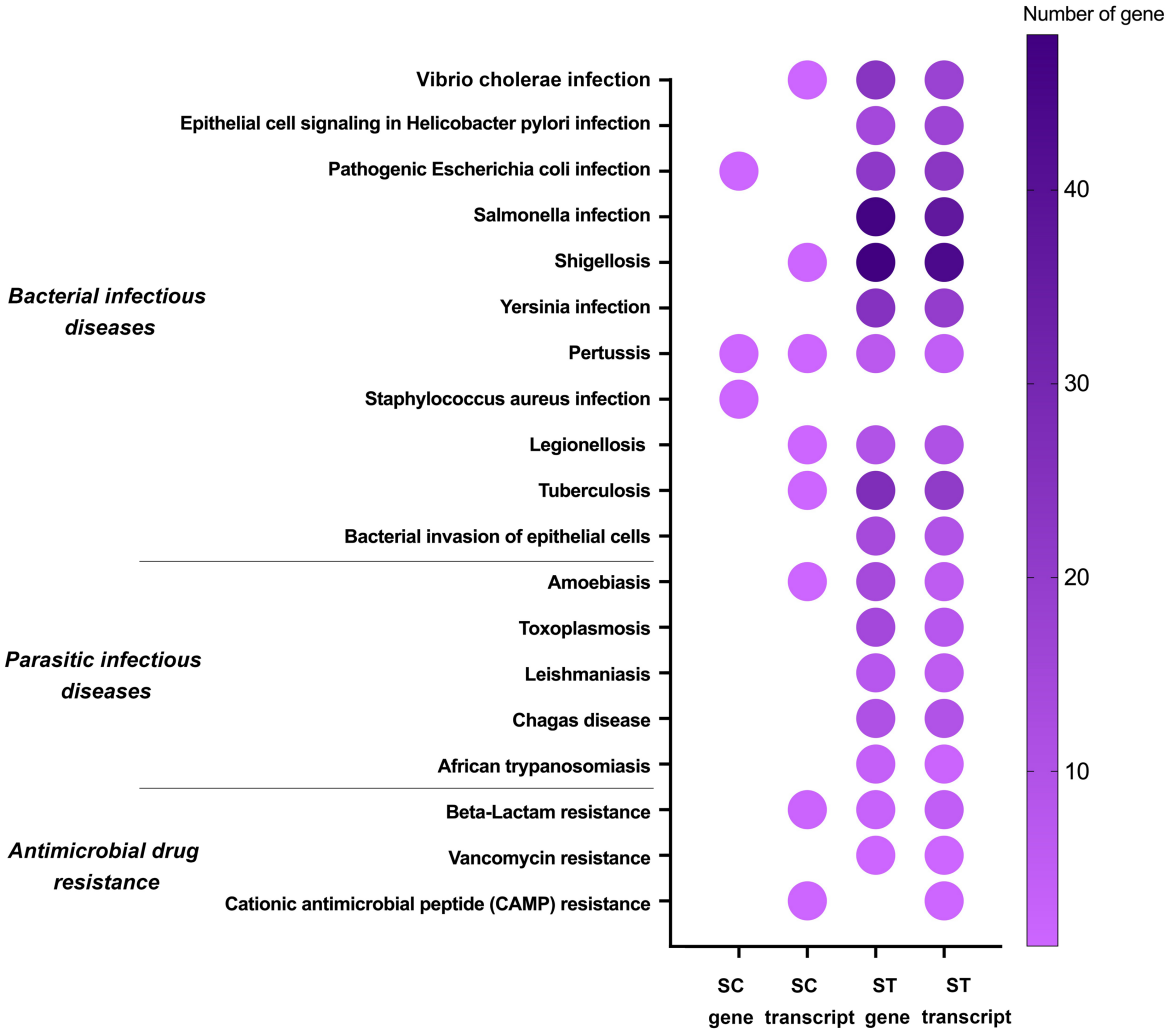
Pathogenicity pathways of genes and transcripts with significant increase in relative abundance after chlorination in SC or ST samples (*p* < 0.05, Quasi-likelihood F-test). Color scale indicates the number of gene or transcript mapped in that pathway. Absence of dot means that no gene or transcript of that pathway was enriched.

**Figure 7:**
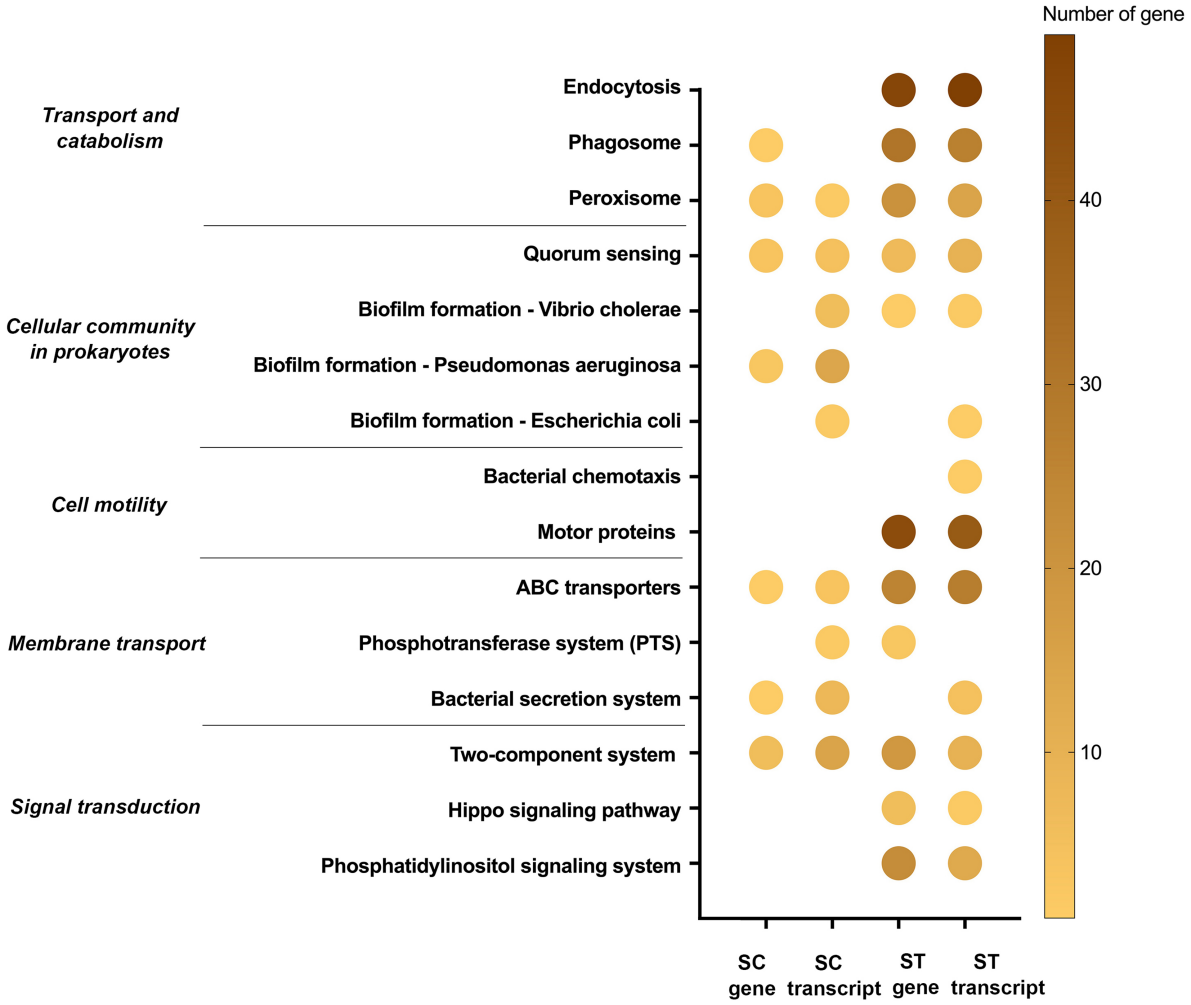
Cellular sensing, signaling and response pathways of genes and transcripts with significant increase in relative abundance after chlorination in SC or ST samples (*p* < 0.05, Quasi-likelihood F-test). Color scale indicates the number of gene or transcript mapped in that pathway. Absence of dot means that no gene or transcript of that pathway was enriched.

**Figure 8:**
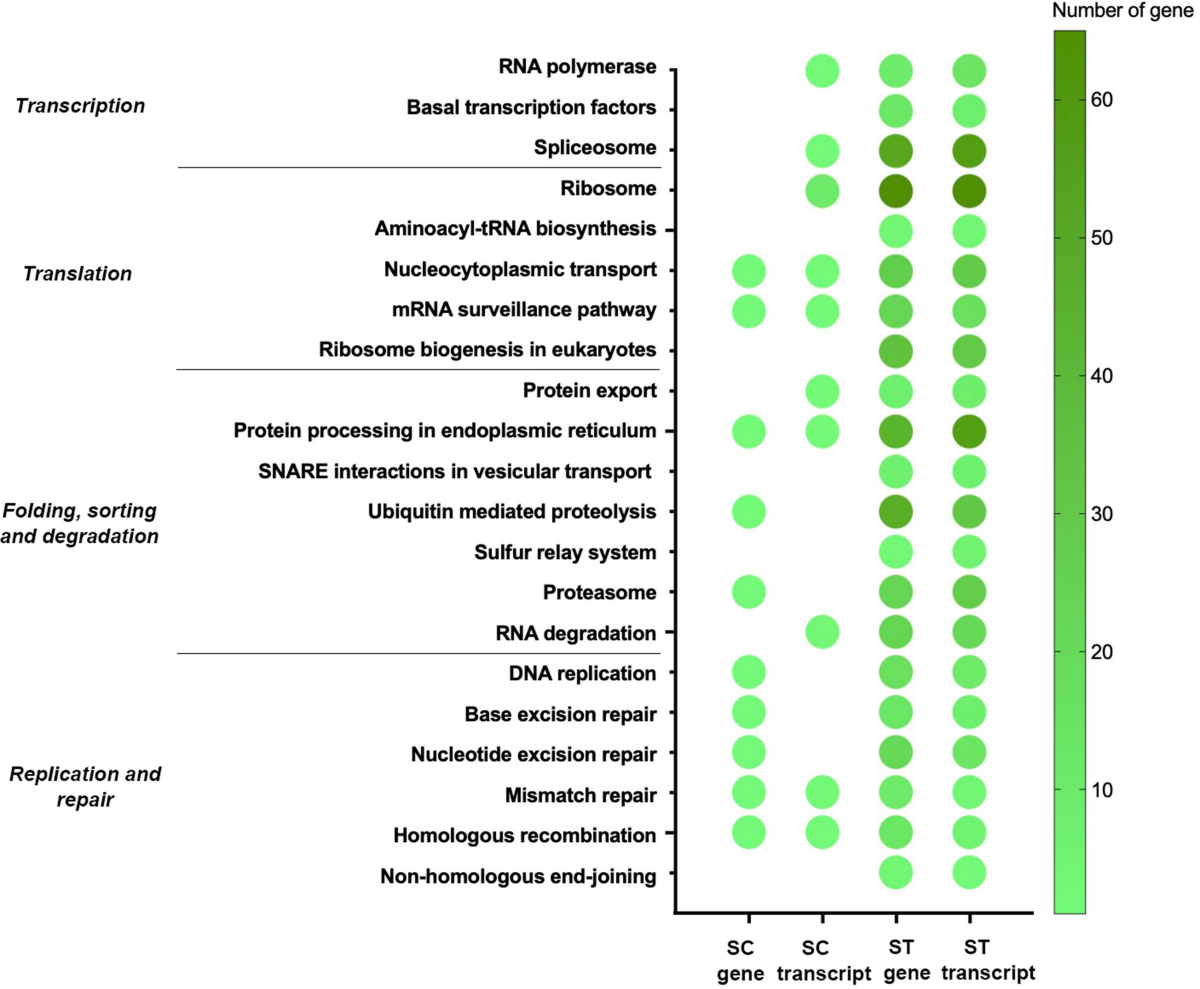
Genetic information processing pathways of genes and transcripts with significant increase in relative abundance after chlorination in SC or ST samples (*p* < 0.05, Quasi-likelihood F-test). Color scale indicates the number of gene or transcript mapped in that pathway. Absence of dot means that no gene or transcript of that pathway was enriched.

The genes and transcripts that had significant increase in relative abundance after chlorination involved in a great variety of pathways (Figures 5–8). Most of the pathways were found exclusively in the genomes and transcriptomes of the ST effluents, the number of mapped genes in each pathway of ST was often higher than that of SC. For metabolism, most of the pathways that were responsible for synthesizing vital cellular components such as carbohydrate, energy, lipid, nucleotide and amino acids had increased abundances of genes or transcripts in both SC and ST samples (Figure 5). For the other categories of metabolism, the microbiomes in each effluent appeared to have specific types of enrich or upregulated pathways. For example, the genes for the biosynthesis of some secondary metabolites such as indole alkaloid, penicillin, clavulanic acid, neomycin, etc. and the degradation of many xenobiotics had increased relative abundances in SC samples. On the other hand, the genes for metabolism of glycan, terpenoids, polyketides and other secondary metabolites were mainly increased in ST samples.

Apart from metabolism, some of the significantly enriched genes and transcripts were associated with microbial pathogenicity, such as bacterial infectious diseases, parasitic infectious diseases and antimicrobial drug resistance (Figure 6). The majority of these pathogenicity-related genes and transcripts were identified in the ST samples. There were 295 genes and 245 transcripts mapped in ST samples, but only 3 genes and 9 transcripts were mapped in SC samples. Among the 11 bacterial infectious diseases, pertussis had increase relative abundances of genes and transcripts in both SC and ST samples. None of the genes related to parasitic infectious diseases showed increased relative abundances in the SC samples, in contrast, the average number of the genes mapped in each pathway was over 10 in the ST samples. Besides, three types of the antimicrobial drug resistance genes and transcripts were also found to have increase in relative abundance, including beta-lactam, vancomycin and cationic antimicrobial peptide resistance.

Some pathways were responsible for cellular sensing, signaling and response (Figure 7). The genes and transcripts involved in peroxisome, quorum sensing, ABC transporters and two-component systems were identified in both SC and ST samples. Transcripts related to the biofilm formation of several types of bacteria were also identified in SC effluents. Some of the pathways were exclusively identified in ST samples, such as endocytosis, bacterial chemotaxis and motor proteins. ST samples had relatively high numbers of genes and transcripts related to endocytosis and motor proteins.

Moreover, many identified genes and transcripts involved in the pathways for genetic information processing (Figure 8). Their functions covered transcription, translation, replication, repair and degradation of genetic materials. Many of them were commonly identified in both effluents, especially for the nucleocytoplasmic transport, mRNA surveillance pathway, protein processing in endoplasmic reticulum, mismatch repair and homologous recombination. It is noted that several types of repair mechanism were significantly increased after chlorination, including mismatch repair, nucleotide excision repair and base excision repair.

On the other hand, the numbers of genes and transcripts with reduced relative abundances after chlorination were much lower than those with increased relative abundances. The total number of significantly reduced genes and transcripts in SC and ST samples was 1937, which was 1,502 lower than those increased. The genes and transcripts that were reduced simultaneously in each effluent were mostly related to oxidative phosphorylation and some types of ABC transporters. The reduction of genes and transcripts related to pathogenicity was negligible, only two genes were found to be responsible for the amoebiasis and *Helicobacter pylori* infection.

### 3.4 MAGs recovered from the effluents

**Figure 9:**
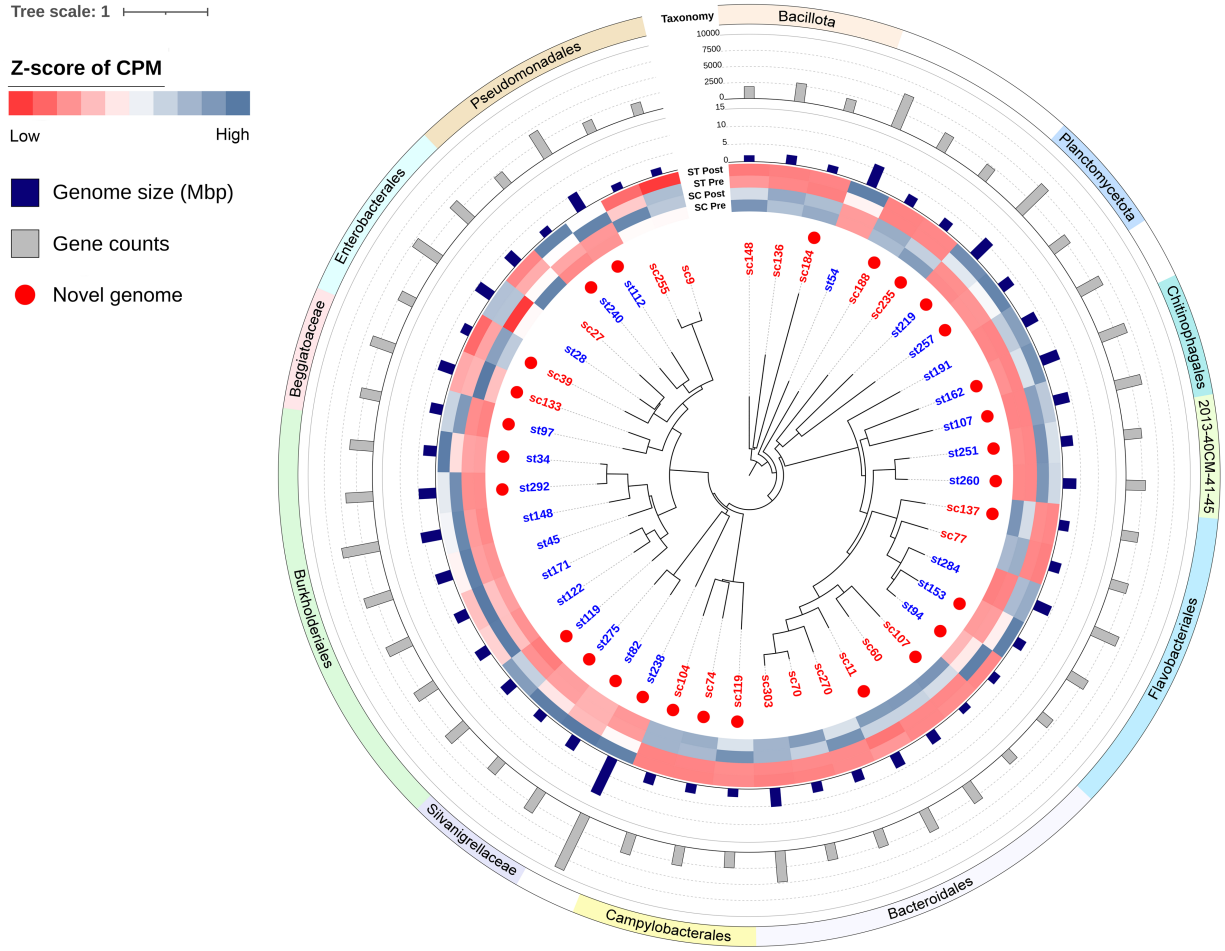
Phylogenetic tree of MAGs. The relative abundances of each MAGs in pre-chlorination (Pre) or post-chlorination (Post) samples of SC and ST are shown in the heatmap. Color scale of the heatmap represented the z-scores of relative abundances of each MAG. CPM stands for coverage per million reads. The genome sizes and gene counts of MAGs are indicated by the bar plots. Red dots indicate the novel genomes which have < 95% ANI with any known MAG in the GTDB. Leaf names in red mean MAGs recovering from SC samples, while those in blue mean recovering from ST samples.

Binning resulted in a total of 46 MAGs with > 90% completeness and < 10% contamination, of which 21 were assembled from SC samples and 25 were assembled from ST samples (Figure 9). MAGs assembled from SC and ST samples were named with the prefixes of sc- and st- respectively. Except 3 MAGs (st28, st171 and sc303), all the other MAGs had a quality score (QS) above 50 [defined as % completeness – (5 × % contamination)]. The N50 of contigs was 18 kbp. Detailed information of each MAG is listed in Supplementary Table S2.

All the 46 MAGs belonged to the Bacteria domain with 18 of them were classified as known species or previously recovered MAGs (Supplementary Table S2). The remaining 28 MAGs did not share an average nucleotide identity (ANI) value of > 95% with any known genomes in GTDB, 6 of them are novel at the genus-level while the others are novel species. Among all the MAGs, only 3 of them (st28, sc136 and sc303) were likely to originate from the human gut microbiome (mash distance < 0.05 to any genome in UHGG catalog, q < 0.05). The sc136 and sc303 had > 60% matched k-mers with at least one genome in UHGG.

These MAGs formed various monophyletic groups in the phylogenetic tree (Figure 9). Many of them consisted of MAGs that were recovered exclusively from one effluent type. For example, the clades of Campylobacterales and Bacteroidales were recovered from SC samples, while the *Silvanigrellaceae* and Burkholderiales were recovered from ST samples. Their relative abundances in the other type of effluent were significantly lower (paired t-test, q < 0.05). It showed that closely related MAGs tended to be recovered from the same effluent. The differences in the relative abundances of MAGs between SC and ST samples also manifested in their corresponding OTUs in the amplicon sequencing data from previous study (Tang and Lau, 2022). Due to the resolution of taxonomy in amplicon sequencing, the OTUs at the genus level were being used for comparison with the known species of MAGs in this study (Supplementary Table S3). Except *Agathobacter*, *Moraxella* and *Rivicola*, MAGs with higher relative abundances in SC (or ST) samples, their corresponding OTUs showed the same. The patterns of variations between SC and ST samples were consistent in both amplicon sequencing and binning results.

Among the recovered MAGs, it is noted that the novel genome st238 was particularly large, with a size of 11.22 Mbp and 9,281 genes. Its genome size ranked among the top 0.07% of all bacterial genomes in GTDB. This novel species belonged to the phylum Myxococcota (Supplementary Table S2). Its closest relative in the database was *JAGNNG01 sp017990795* (GCA_017990795.1), sharing an ANI value of 77.5%. Among all the novel genomes in the tree, the closest relatives were the two *Undibacterium* of Burkholderiales (st34 and st292), which had an ANI value of 71.10%.

**Figure 10:**
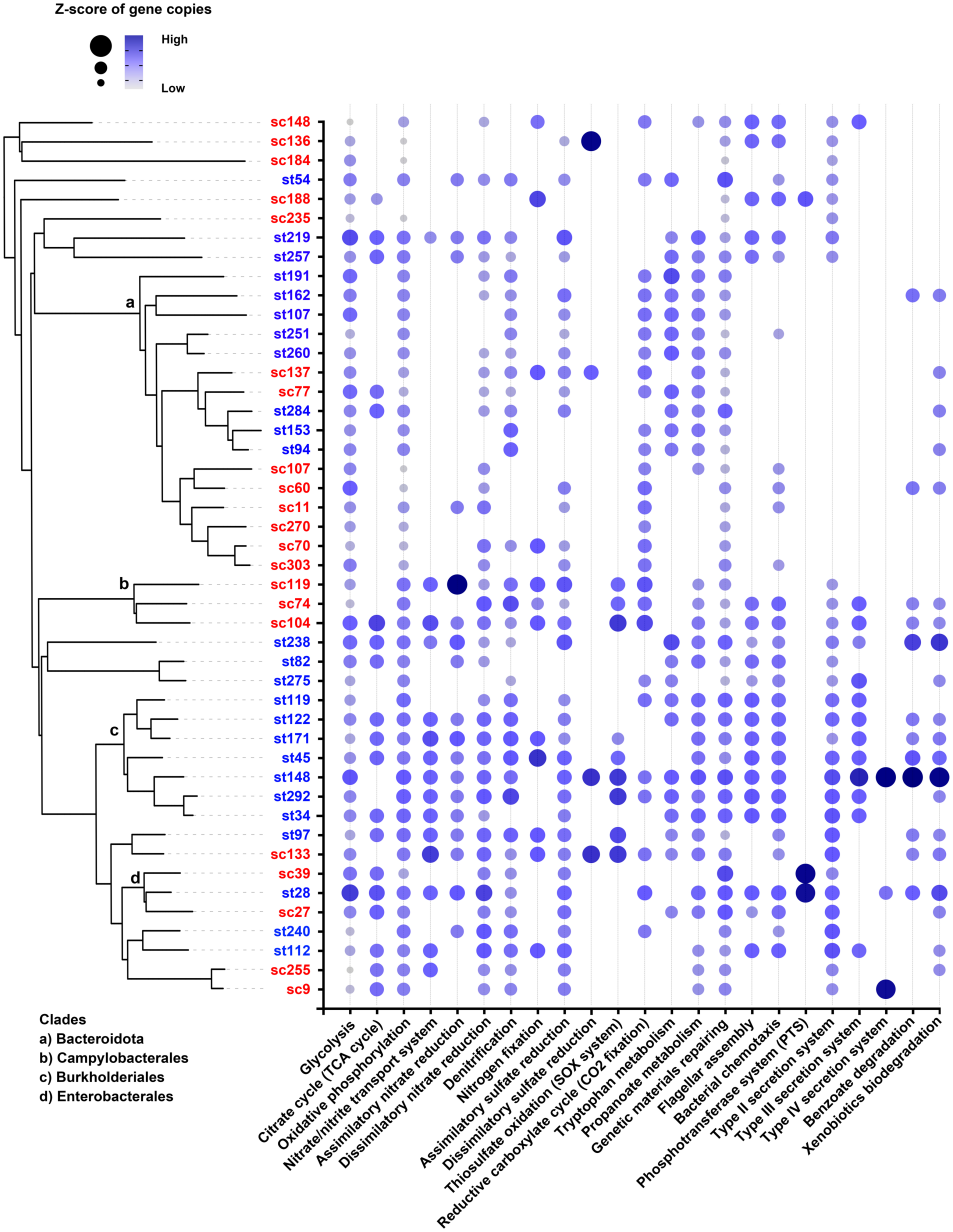
Functional profiles of MAGs. The dot size and color represent the range of z-scores of the gene copies of each functional pathway. Absence of dot means that no gene of the pathway was found in that MAG. The clades of Bacteroidota, Campylobacterales, Burkholderiales, and Enterobacterales are indicated in the phylogenetic tree.

MAGs under the same order shared similar characteristics in their functional gene profiles (Figure 10). For examples, Burkholderiales had high copy numbers of genes related to nitrate uptake and reduction, and Campylobacterales possessed genes involved in thiosulfate oxidation (SOX system). Some genetic functions were only present in a few MAGs. For example, genes responsible for phosphoenolypyruvate sugar phosphotransferase system (PTS) were particularly found in two Enterobacterales (sc39 and st28), and the genes responsible for type IV secretion system were only found in st148, st28 and sc9. On the contrary, certain types of MAGs lacked specific functions that were ubiquitous among other MAGs. For example, the Bacteroidota (including Chitinophagales, *2013-40CM-41-45*, Flavobacteriales and Bacteroidales) did not possess any genes for type II secretion (T2S) system.

## 4 Discussion

### 4.1 Comparison of genomic and transcriptomic profiles between effluents

The microbiomes of SC and ST effluents had very distinct genomic and transcriptomic profiles as shown by the clear segregation between two effluents in the MDS plots (Figure 1). The relative abundances of many mapped genes and transcripts were significantly different between two effluents (Figures 3C,D). In the previous study of Tang and Lau (2022), amplicon sequencing results also revealed substantially different taxonomic compositions between SC and ST effluents, probably due to the operational differences of two sewage treatment works. Since SC received seawater-based sewage influents for primary treatment while ST received freshwater-based influents for secondary treatment, the salinity and nutrient levels of the effluents were highly different, establishing two contrasting taxonomic profiles. Factors including salinity, DO, BOD₅, TSS and TN appeared to be associated with the variances in genetic functions and activities of the effluent microbiomes, suggested by their significant correlations with the genomic and transcriptomic profiles (Table 1). Nonetheless, as the physicochemical parameters might be co-varied with each other and gradients of concentrations were absent, it cannot be concluded that certain factors as dominant or affecting the microbiomes individually. Studies on extensive types of effluents or experiments with single varying factor will be needed to isolate the effect of each parameter.

Our findings showed that the type and extent of alterations in genomic and transcriptomic profiles upon chlorination differed by effluents. Most of the genes and transcripts with significant changes in relative abundances after chlorination were observed exclusively in either effluent (Figure 4). This can be attributed to the large discrepancy of the taxonomic compositions of two effluents. It is because different types of microbes could exhibit different sensitivity and stress responses to chlorination. Also, it is observed that the number, proportion and fold change of altered genes and transcripts in ST effluents were higher than that in SC effluents. One reason might be due to the higher total number of genes and transcripts mapped in the ST effluents, which were around 3,000 more than the SC effluents. The observation also suggested that the microbial genetic functions and activities in the different effluents might have varying susceptibility to the influence of chlorine stress. Some microbiomes might be more sensitive to the chlorination process, reflected by great alterations of their gene abundance and expression.

The significant differences of the altered genes and transcripts in different effluents indicated that the selection of chlorination on the genotypes of microbiomes might not be the same among effluents. Different microbiomes would be subjected to different scale of effect under chlorination in terms of the genetic potential and activities. It implied that the chlorine disinfection outcomes varied among effluents, including the viability, genotypes, functionality and activities of the remanent microbiomes in chlorinated effluents, and hence the potential consequences associated with the effluents after discharge.

### 4.2 Implications of the altered functional pathways after chlorination

To further deduce the survival strategies and responses of different effluent microbiomes under chlorination, we examined the alterations in the relative abundances of functional pathways in the two effluents. Most of the significantly changed genes and transcripts had increased relative abundances after chlorination. These enriched genes and transcripts were responsible for a great variety of functions, which offered many advantages for microbial survival under chlorination or acted as responses to counteract the chlorine stress (Figures 5–8).

Among all the functional pathways with significantly increased relative abundances after chlorination, many of them belonged to metabolism (Figure 5). The metabolic pathways of essential components, including carbohydrate, energy, lipid, nucleotide and amino acids, had increased relative abundances of genes and transcripts in both SC and ST effluents. It is known that RCS have fast and potent oxidizing reactions with biomolecules such as sulfur-containing amino acids, lipids and nucleic acids. This will damage membrane compounds, disrupt ATP production, causes protein fragmentation and aggregation, as well as the dissociation of DNA double-strands (Nizer et al., 2020). Many of these reactions are irreversible. The loss of these molecular compounds will hinder cell growth and reproduction, eventually leading to cell death. This study showed that microbes equipped with greater potential of synthesizing these compounds would be more likely to survive the chlorination. The metabolism of these compounds might have helped maintain the essential physiological functions for surviving the chlorine stress. Furthermore, the higher relative abundances of transcripts suggested that the microbes might activate these functional pathways after encountering the chlorine stress to compensate the loss of biomolecules. As observed in the microcosm experiments of previous study, the effluent communities showed regrowth in seawater after release from sublethal dosage of chlorine (Tang and Lau, 2023). It might be benefited from the active metabolism as it provided energy and building blocks for the recovery and regrowth of microbial communities when the environmental conditions become stable again.

Apart from the essential components, the remanent microbiomes in chlorinated effluents also showed higher relative abundances of genes and transcripts for the metabolism of secondary metabolites and xenobiotics (Figure 5). Although secondary metabolites are not vital for microbial growth, many of them have important ecological functions such as interspecies competitions or symbiotic communications (O’Brien and Wright, 2011; Sansinenea and Ortiz, 2011; Thapa and Grove, 2019). While xenobiotics are often originated from products such as drugs, personal care products, industrial additives and other synthetic compounds, some of them are discharged into urban sewage treatment facilities by human excretion or disposal (Mishra et al., 2021; Štefanac et al., 2021). They are rather difficult to be removed during sewage treatment, but some microbes are able to catabolize these xenobiotics as nutrient sources to fuel their growth and metabolic activities (Mishra et al., 2021). Therefore, these metabolic pathways would provide microbes with more competitive and adaptive advantages under stressful conditions. It is noted that many of the pathways were specific to one of the effluents, suggesting that the remanent microbiomes had different preferences on the types of secondary metabolites and xenobiotics being synthesized or used.

On the other hand, the abilities to sense and respond to external stimuli were important for the microbes to endure and resist the chlorine stress, as shown by the significantly increased relative abundances of genes and transcripts in chlorinated effluents (Figure 7). The relative abundances of pathways related to two-component systems were increased in both effluents. This type of pathway has been reported for regulating stress responses to counteract oxidative stress, including the chlorine-induced oxidative stress (Ahn et al., 2012; Tatsuno et al., 2014; Bensig et al., 2022; El Hajj et al., 2022). Some pathways related to cell motility including the motor proteins and bacterial chemotaxis were increased in the microbiomes of ST. These pathways were particularly found in the MAGs of *Silvanigrellaceae* and Burkholderiales recovered from the ST effluents as well (Figure 10). They can assist microbes in nutrient acquisition and avoiding unfavorable environment (Colin et al., 2021). Meanwhile, the remanent microbes had higher relative abundances of genes and transcripts related to cooperation with the microbial community, such as quorum sensing and biofilm formation. It allowed microbes to relay information and coordinate behavior of the community to tackle adverse environmental conditions and establish a relatively stable microniche for better adaptation (Miller and Bassler, 2001; Flemming and Wingender, 2010). These observations showed that chlorination might be a selective force for microbes with greater potential or activities in sensing and reacting to external conditions, as well as community communication and cooperation. These microbes had higher chance to survive the chlorination and adapt to the environment after discharge.

Since RCS react with proteins and nucleic acids rapidly, materials for genetic information processing such as DNA, RNA and proteins might have severe damage during chlorination. Therefore, many genes and transcripts related to the repairing of genetic materials and degradation of damaged proteins had increased relative abundances after chlorination in both effluents (Figure 8). Moreover, the microbiomes had higher relative abundances or upregulation of genes involved in transcription or translation processes as they were crucial for microbes to adjust different cellular processes and maintain homeostasis. It showed that though chlorine might have caused a wide range of cellular damage, instead of conserving energy, the microbiomes gave higher priority to activate various cellular processes through protein synthesis, which demonstrated their resilience in the face of chlorine stress.

For both sewage treatment works, the chlorinated effluents will be discharged directly to the coastal seawaters. The impact of the remanent microbiomes to the ecosystem and public health is of particular concern. Our results showed that many of the increased genes and transcripts in chlorinated effluents were associated with waterborne diseases such as *Vibrio cholerae* infection, S*almonella* infection, shigellosis and legionellosis (Threlfall, 2002; Koutsotoli et al., 2006; Lutz et al., 2013; Falkinham, 2020) (Figure 6). Moreover, the microbiomes possessed several types of resistance genes against antimicrobial drugs such as vancomycin and beta-lactam. It is noted that nearly all the pathogenicity-related genes and transcripts had significant increase after chlorination in the ST effluents, suggesting that some chlorinated effluents might have higher risks of causing diseases or contain higher numbers of antimicrobial resistance genes. It will become a public health threat if the persisting microbes are infectious and transmitted through contaminated water or food to human. The antimicrobial resistance genes could also spread to other bacteria in the ecosystem via horizontal gene transfer, rendering antibiotic treatments less effective (Von Wintersdorff et al., 2016). This is particularly relevant to the discharge of effluent as aquatic environment is a known hotspot for horizontal gene transfer (Pereira et al., 2018).

With reference to the findings in previous studies (Tang and Lau, 2022, 2023), in which all the effluent samples had been examined using cultivation, qPCR and 16S rRNA gene amplicon sequencing coupled with or without propidium monoazide (PMA) treatment, a considerable amount of viable cells existed in the chlorinated effluents (5–11 log_10_ 16S rRNA gene copies ml^−1^), despite the substantial reduction of culturable fecal indicator bacteria (FIB). Some clinically important pathogenic species including *V. cholerae* were found viable after chlorination. There were high concentrations of viable pathogens in the chlorinated effluents (over 8 log_10_ 16S rRNA copies ml^−1^). These results support the findings of pathogenicity-related genes and transcripts from the metagenomes and metatranscriptomes in this study, showing that the chlorinated effluents were still carrying many viable pathogens with active gene transcriptions. Furthermore, microcosm experiments were conducted in previous study to trace the survival of bacteria by simulating the discharge of chlorinated effluents into coastal seawater. A rapid increase of the proportion of viable SC communities were shown after 24 h of incubation in seawater, showing the resilience of chlorinated effluent communities after discharge. This suggested the ecological risks of discharging the chlorinated effluents into the receiving environment. Although regulatory agencies apply culture-based enumeration of FIB for monitoring the risks of fecal pollution in disinfected effluents, this method was proven to be ineffective for detecting the vast populations of viable but non-culturable (VBNC) cells in the effluents (Tang and Lau, 2022, 2023). These VBNC cells are non-reproductive yet structurally and metabolically intact, which lost the capability to form colony on culture media but retain the ability to resuscitate in certain conditions (Pinto et al., 2011). The limitation of cultivation assays in detecting VBNC FIB weakens the performance of FIB as a proxy of detecting viable pathogens. Therefore, current FIB paradigms cannot effectively reveal the pathogenicity of effluents. For exploratory purpose, metagenomics and metatranscriptomics provide information about the presence and expression of pathogenicity-related genes in the microbial communities, which establish basis for the potential genes or transcripts as the biomarkers of the risks associated with effluents. Further studies on the applicability of alternative robust monitoring approaches such as PMA-qPCR for specific genomic indicators would be valuable for assessing the ecological impact of effluents after disinfection.

In this study, none of the genes and only a few transcripts were commonly reduced in both effluents. This suggested that the chlorine-sensitive genes were different between effluents. It might be due to the large disparity of microbial compositions in two effluents such that microbes with distinct genomic profiles were reduced or exhibited different responses under chlorine stress. Also, the reduction of genes or transcripts in various types of functional pathways was far less than those that were increased, implying that microbes with higher capability or activities in cellular processes were more likely to survive the chlorination. The genes and transcripts with significantly increased relative abundances after chlorination gave insights into the cellular functions that enhanced microbial survival under chlorination. Microbiomes in different effluents exhibited variations on the types of pathways, but in general these pathways equipped microbes with stronger capability to acquire nutrients and energy, repair damaged cellular components, sense and react to chlorine stress. They also provided adaptive advantages for the microbial community to recover after the removal of chlorine stress. Furthermore, the genomic potential and activities of remanent microbiomes could help us predict their behavior and influence after discharge to the environment, including the risks associated with pathogenic diseases. These potential risks might vary across different effluents. Therefore, the discharge standards of the microbiological quality of disinfected effluents would need to be adjusted accordingly. Nonetheless, the findings in current investigation were based on the gene abundance and expression of the microbiomes from sequencing data, further experimental validations such as qPCR for target genes, infectivity assays and phenotypic testing of antimicrobial resistance would enhance the authenticity of the observations and interpretation. Also, as the microbiomes in the discharged effluents can be affected by many other factors in the receiving environment such as microbial interaction, dilution effect and bioaccumulation, the actual impact of discharged effluents on the ecosystem and public health would need to be confirmed by extensive field studies.

### 4.3 Characteristics of the recovered MAGs

In agreement with the contrasting taxonomic compositions of two effluents, distinct types of MAGs were recovered from each sewage treatment work. MAGs belonging to the same order or having closer evolutionary relationship tended to be recovered from the same effluent (Figure 9). These MAGs shared some genetic functions which aligned with the conditions of the respective effluent. For instance, Burkholderiales recovered from ST effluents contained genes for nitrate transport system and assimilatory nitrate reduction, which were absent in most of the MAGs found in the SC samples (Figure 10). This matched with the observation of high nitrate concentrations in ST effluents (3–4 times higher than in SC effluents), as ammonia or ammonium were converted to nitrate by the biological oxidation in secondary treatment. It might be favorable for bacteria that could acquire and utilize nitrate as nitrogen source for growth through nitrate transport and reduction.

Although these MAGs were recovered from municipal sewage effluents, most of them were not originated from human gut microbiome. None of the novel MAGs was likely to be originated from human gut. Low proportions of human gut microbial MAGs recovered from effluent samples were also observed in other studies (Hendriksen et al., 2019; Jespersen et al., 2023). The identification of novel human gut microbial genomes in sewage samples using metagenome binning approach appeared to be limited. However, the low number of recovered human gut MAGs did not necessarily represent the abundances of fecal-origin microbes in the effluents because only a small number of MAGs could be recovered from the effluents. Taxonomy annotation or sequence alignment to databases with fecal source records would be useful for the estimation of the abundances of fecal-origin microbes in effluents.

In this study, all the filtered MAGs were Bacterial, none of them were Archaeal. It might be due to the large disparity of their proportions in the effluents. The average percentages of Bacteria over the total population in the metagenomes were 77.176 ± 15.835%, while that of Archaea were only 0.013 ± 0.008%. The two most abundant phyla recovered from effluent samples were Bacteroidota and Pseudomonadota, 16 out of the 46 MAGs were identified for each phylum. It is noted that all the Bacteroidota including the 9 novel members did not possess any genes for T2S system (Figure 10). This agrees with current literatures that no evidence has been shown for potential T2S system in Bacteroidota, despite the ubiquity of T2S in Gram-negative bacteria (Cianciotto, 2005; Cianciotto and White, 2017).

In conclusion, this study highlights that microbiomes in different effluents varied substantially in the taxonomic compositions, genomic potential and activities with or without going through chlorine disinfection. By demonstrating that these microbiomes did not respond to chlorine disinfection uniformly, this study underscores the necessity of integrating genomic and transcriptomic insights into the setting of effluent discharge standard. As seawater toilet flushing is an emerging alternative for coastal cities to conserve freshwater resources, the investigation of seawater-based effluents in parallel with freshwater-based effluents with similar local context would be valuable for the tailoring of disinfection regimes and discharge standards. Nonetheless, the discrepancy of the alterations in genomic and transcriptomic profiles of two effluents in this study was not only restricted to the difference in salinity, but could also be attributed to various properties of the sewage treatment works, including the treatment stage, organic loads, operational conditions and so on. As limited by different geographic locations and distinct operational settings, the changes of specific genes or transcripts under chlorination in certain type of effluent may not be transferrable to other urban systems or treatment plants with similar properties. It is sensible to evaluate the effects of disinfection specifically for the microbiomes established in each effluent, in order to have a better assessment of the potential impact of effluent discharge on the environment and public health.

## Data Availability

The metagenomic and metatranscriptomic sequencing data were deposited at NCBI Sequence Read Archive (SRA) with accession no. of PRJNA1156557.

## Footnotes

^1^ https://bioinformatics.psb.ugent.be

^2^ https://www.graphpad.com/scientific-software/prism/

^3^ https://itol.embl.de
