# Supplementary material for: Varying effects of chlorination on microbial functional repertoire and gene expression in contrasting effluents
Source: Front Microbiol. 2025 Jun 18;16:1593147. doi: 10.3389/fmicb.2025.1593147 (PMC12213577; doi:10.3389/fmicb.2025.1593147)
Supplement: Supplementary file 1 [file Data_Sheet_1.docx]

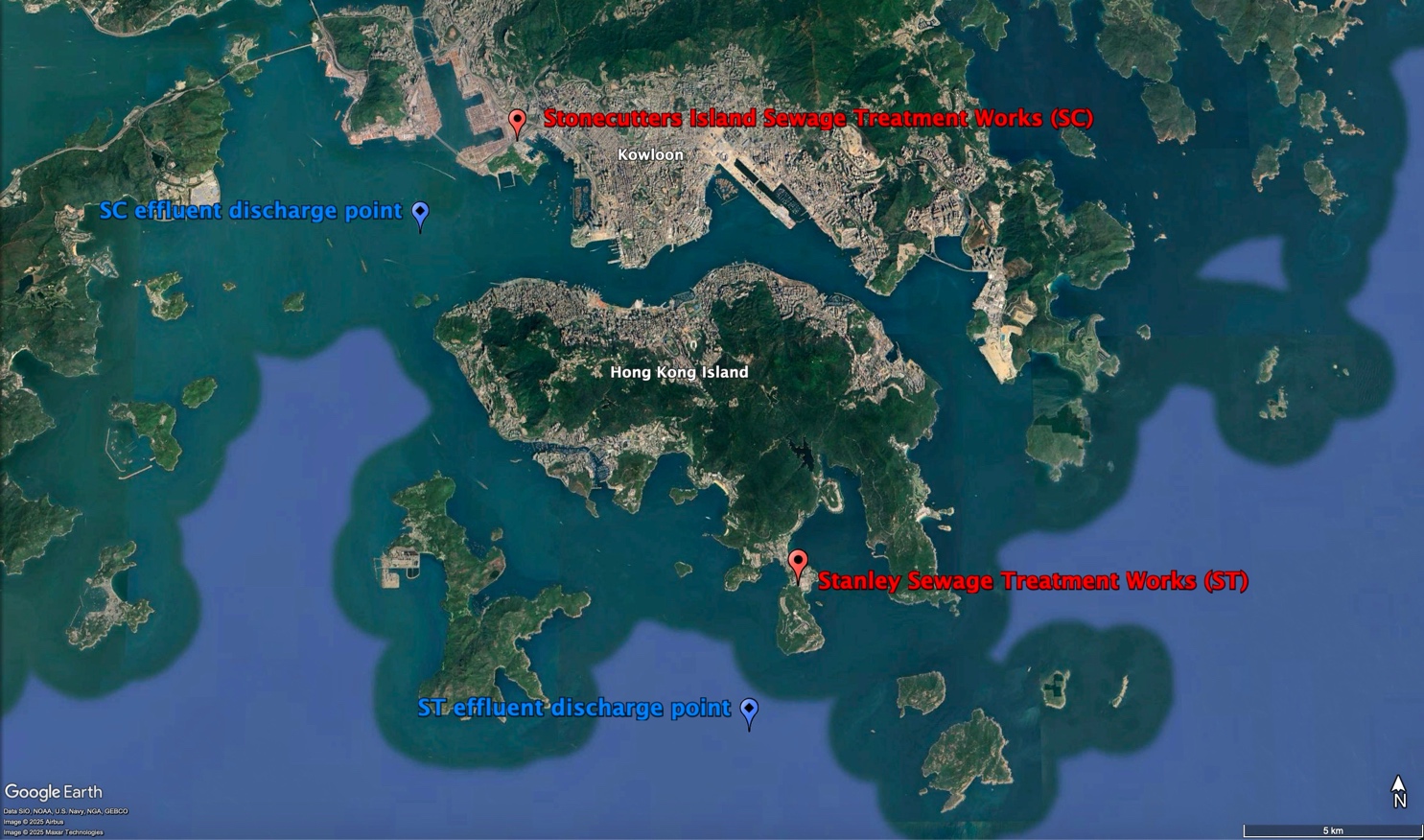


**Figure S1.** Locations of Stonecutters Island Sewage Treatment Works (SC) and Stanley Sewage Treatment Works (ST) in Hong Kong. This map indicated the locations of SC (22.326809°, 114.138946°) and ST (22.211563°, 114.217026°), together with their approximate discharge points in the coastal seawater. Figure is generated by using Google Earth Pro (7.3.6.10201).


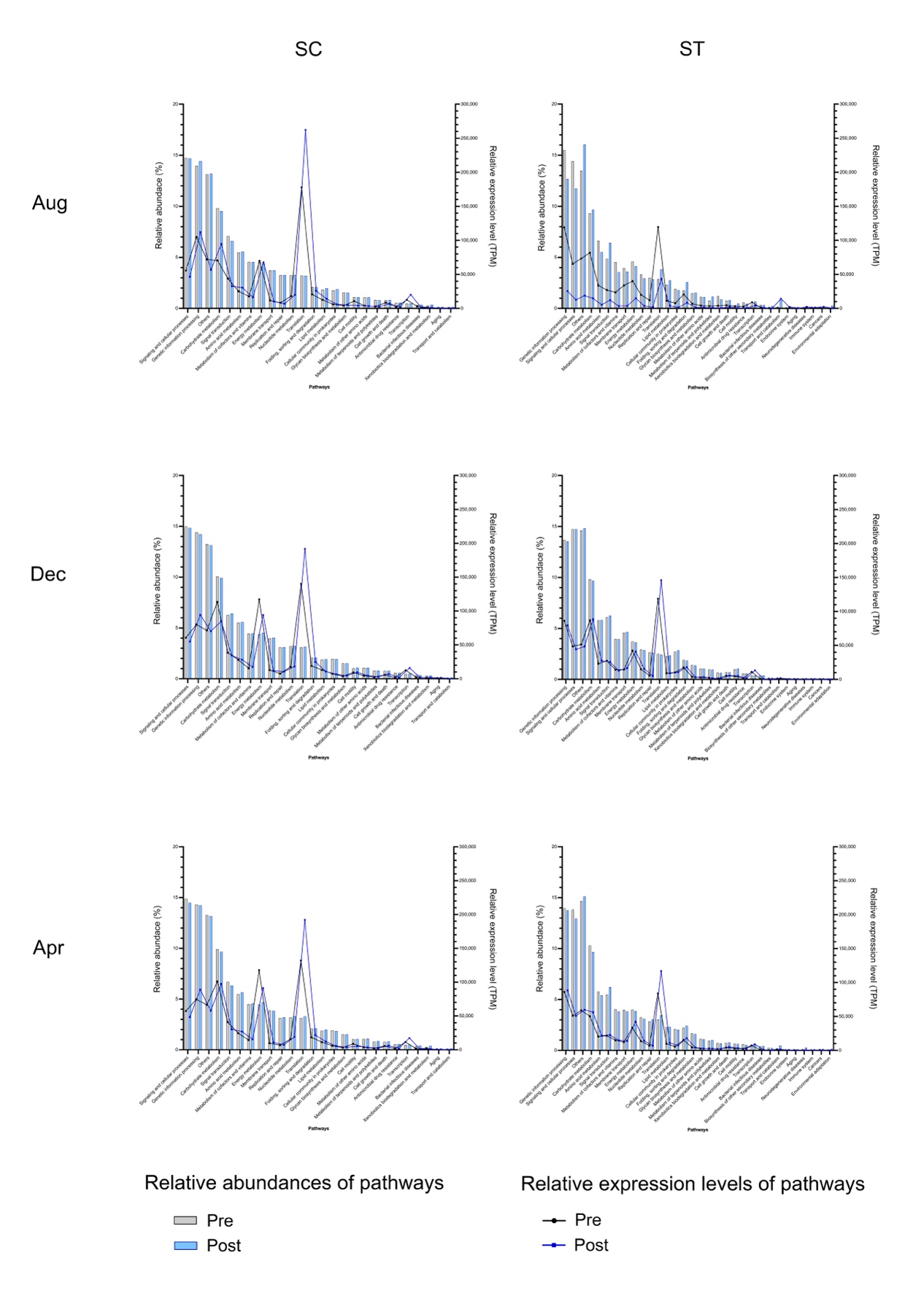


**Figure S2.** The relative abundances and expression levels of functional pathways in the samples collected in different months. Bars indicate the percentages of relative abundances, while lines indicate the transcripts per million (TPM) of relative expression levels. Pre and Post represent samples before and after chlorination respectively.
